# Medicinal plants in a changing climate: understanding the links between environmental stress and secondary metabolite synthesis

**DOI:** 10.3389/fpls.2025.1587337

**Published:** 2025-06-13

**Authors:** Devesh Jangpangi, Babita Patni, Vaishali Chandola, Sudeep Chandra

**Affiliations:** High Altitude Plant Physiology Research Centre, HemvatiNandan Bahuguna Garhwal University, Srinagar, Uttarakhand, India

**Keywords:** abiotic stress, climate change, medicinal plants, secondary metabolites, phytochemical biosynthesis, omics technologies, plant adaptation, epigenetic regulation

## Abstract

Environmental stresses, including temperature extremes (cold and heat), elevated CO_2_, and ozone, significantly influence the production of plant secondary metabolites (PSMs). These environmental factors can lead to significant changes in the morphology, physiology, and biochemistry of plants. Natural resources, especially medicinal plants, have been used for centuries for their healing properties. PSMs, compounds with unique characteristics, often accumulate in response to stress, playing a crucial role in plant adaptation and stress tolerance. While environmental variables like temperature, light, water availability, humidity, CO_2_, and mineral nutrition are known to impact plant development and PSM synthesis, research on the effects of climate change on medicinal plants is limited compared to other commercial crops. This review examines the impact of various environmental factors on PSM synthesis in medicinal plants and identifies key knowledge gaps. We highlight the need for further research in this area and suggest potential directions for future studies to better understand and potentially manipulate the relationship between climate change, environmental stress, and the production of therapeutically valuable PSMs.

## Graphical Abstract

The graphical abstract showing the impact of different abiotic stresses on plant based secondary metabolites. The adaptation strategies to mitigate adverse effects and sustain medicinal plant resources.

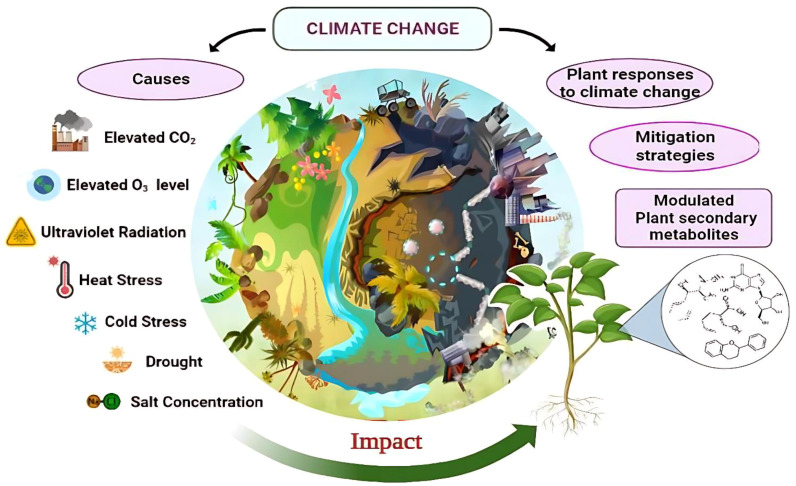

## 1 Introduction

Ecological communities and geographical distributions of plant species worldwide have been suffering the consequences of climate change (Applequist et al., 2020). The Fifth Assessment Report (AR5) of the Intergovernmental Panel on Climate Change (IPCC) projected that the global mean temperature, relative to preindustrial levels, is likely to increase by 1.5°C to 4°C by the end of the twenty-first century. Additionally, the report highlighted the likelihood of experiencing intense and unpredictable weather events in the future (Wu et al., 2018).

Climate change has notably affected medicinal plants, both cultivated and wild, with observable impacts on their growth, distribution, and overall health. A prompt and specific approach to studying these changes is crucial, especially when it comes to the gathering of vital secondary compounds that are essential components and significantly contribute to the preservation of human well-being.

Plant secondary metabolites (PSMs) are produced as byproducts of primary metabolic processes in plants (Khare et al., 2020). These chemical compounds can be volatile or non-volatile and produced through various metabolic pathways in plants, the PSMs have wider applicability including helping plants to adjust to various environmental conditions through affecting the physiological and ecological functioning of plants (Hussein and El-Anssary, 2019). Research studies on medicinal plants in relation to climate change are notably scarce and limited in comparison to other commercial crops.

Medicinal plants serve as promising reservoirs of nutraceuticals and bio-molecules, and it is time that this category of plants cannot be ignored (Harish et al., 2012). This review article thus aims to understand how climate change could affect the production of secondary compounds in medicinal plants. Medicinal plants, known for their diverse array of secondary metabolites with therapeutic properties, are particularly sensitive to a changing climate. The present review explored how changes in temperature, precipitation, elevated CO_2_ levels, and other climatic variables influence the biosynthesis of secondary metabolites, the gene expression, enzyme activities etc. The findings of this review may help policymakers and researchers to develop strategies for mitigating the impacts of climate change, including conservation efforts, cultivation practices, and biotechnological interventions.

## 2 Materials and methods

Seven different publication databases including Scopus, PubMed, Web of Science, Research Gate, EBSCO Green FILE, and Google Scholar were searched for the literature using the keywords “Climate change”, “Environmental stress”, “Plant adaptation”, Gene modification”, “Plant ecophysiology”, and “Secondary metabolite synthesis” etc. The articles obtained from these searches were scrutinized in the context of the medicinal plants. Further selected articles were studied and analyzed for a comprehensive review reporting how changing climatic conditions affecting the physiology and biochemistry of medicinal plants.

## 3 Plant secondary metabolites synthesis

Plant secondary compounds are involved in various defense functions, like protection against UV radiation, inhibiting enzymes, acting as antioxidants, and producing pigments. Secondary metabolites are organic compounds synthesized by plants that do not directly participate in their growth, development, or reproduction; instead, these compounds help plants to sustain in harsh climatic conditions (Hu et al., 2018).

There are about 30,000 compounds that belong to the class of Terpenoids (Mabou et al., 2021), around 8,000 phenolic compounds (Vuolo et al., 2019), and 21,000 compounds are alkaloids. The synthesis of secondary metabolites utilizes various metabolic pathways originating from primary metabolites. These metabolites can be categorized into two types based on their chemical composition:nitrogenous and non-nitrogenous compounds. Nitrogenous compounds are further classified as amines, amino acids, glucosinolates, non-proteinogenic, alkaloids and cyanogenic glycosides. The categorization of alkaloids includes several different types, such as proto alkaloid, cyclopeptide alkaloid, polyamine alkaloid, free alkaloid and pseudo alkaloid. Alkaloids are mainly composed of amino acids, which are considered the fundamental building blocks for their formation (Wink, 2010).

However, non-nitrogenous compounds, such as polyacetylenes and phenolics, are divided into four main categories (like arylpyrones,styrylpyrones, terpenoids and polyketides (Guerriero et al., 2018). Phenolic compounds are formed through two main pathways: the shikimic acid pathway and the malonate acetate pathway. These compounds are broadly classified into two major types: phenolic acids and phenylpropanoids (Naikoo et al., 2019). The phenylpropanoids are of eight types i.e. cinnamic aldehydes, suberin, lignans, flavonoids, stilbenes-coumarins, hydroxyl-cinnamic acid and lignin. The flavonoids compounds are divided into categories such as flavonols, isoflavones, flavones, flavanones, and anthocyanins.

Terpenoids, another category of secondary metabolites, are derived from a compound called isopentenyl diphosphate (IPP) that contains five carbons and serves as a precursor (Thirumurugan et al., 2018). The terpenoids are synthesized from the mevalonate pathway or the methyl erythritol phosphate pathway (Bartram et al., 2006). Terpenoids are classified on the basis of the presence of isoprene units like monoterpenes, triterpenes (which include sterols), diterpenes (such as gibberellins), sesquiterpenes (like ABA), and tetraterpenes (like Carotenoids).

## 4 Effect of climate change on secondary metabolites production

**Figure 1:**
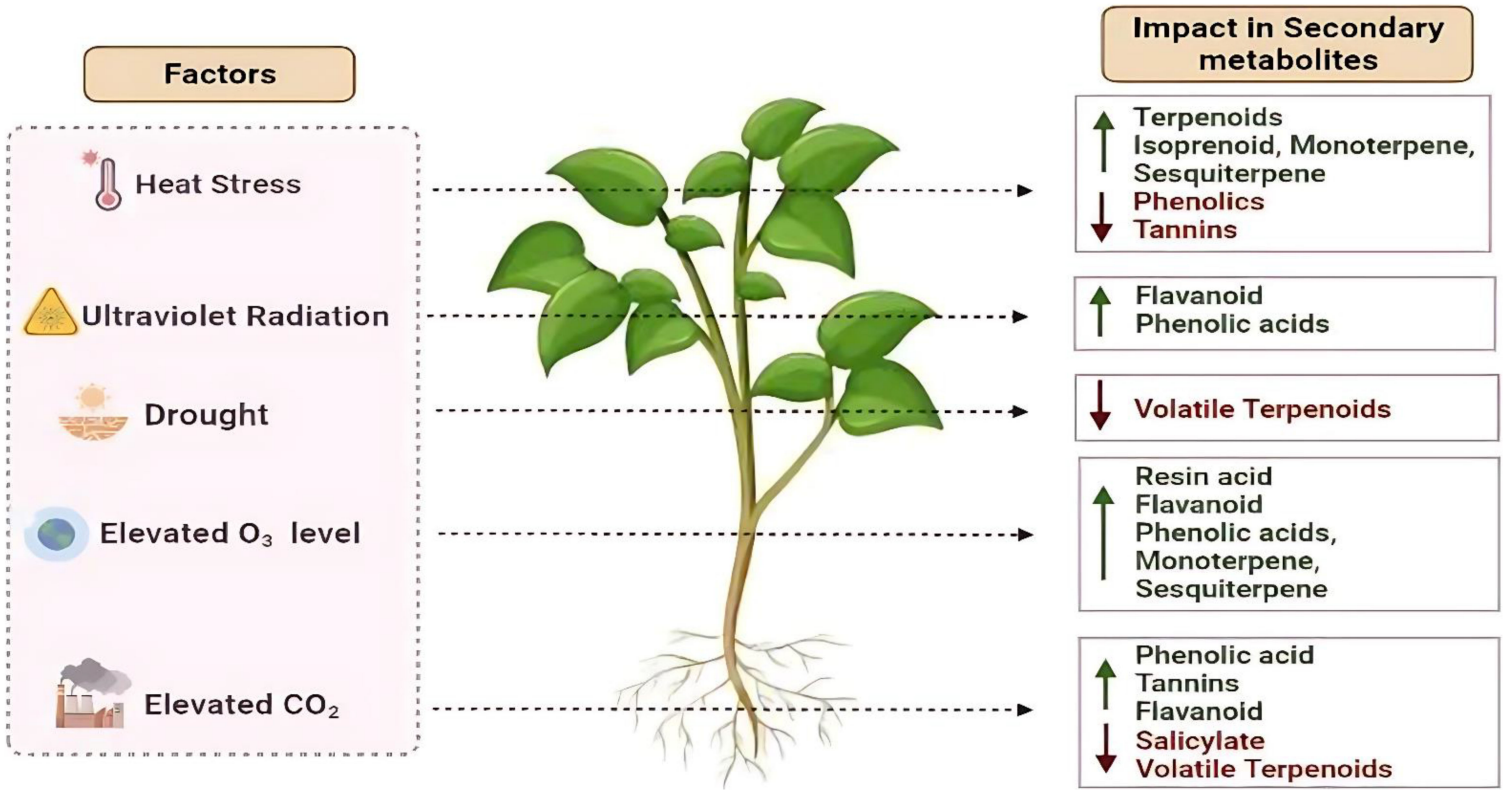
Schematic representation showing the impact of different abiotic stresses (e.g., drought, temperature extremes, salinity, elevated CO_2_, ozone, UV-B) on the production levels of various plant secondary metabolites. Upward and downward arrows indicate increases or decreases in metabolite levels, respectively.

The shifting climate patterns are causing medicinal plants to experience environmental stresses, impacting their growth, development, and production of secondary metabolites (Pant et al., 2021; **Figure 1**). Increased levels of carbon dioxide (CO_2_) can impact plant secondary metabolism since CO_2_ is crucial for photosynthesis and the overall growth of plants (Jamloki et al., 2021). Another factor elevated temperatures also leads to heat stress in plants, affecting their structure, functions, and biochemical processes. The previous studies showed both increased and decreased amounts of plant secondary metabolism under simulated climate change (Pant et al., 2021; Chandra et al., 2022a, 2022). Other abiotic stresses, such as drought, salinity, exposure to heavy metals, and nutrient deficiencies also affect plant secondary metabolism (Punetha et al., 2022). The abiotic stresses induce the activation of genes responsible for the synthesis of secondary metabolites, antioxidants, osmolytes, and phytohormones (Mahajan et al., 2020; Akula and Ravishankar, 2011). Both internal developmental and external environmental factors influence the production of secondary metabolites in medicinal plants (Li et al., 2020). Developmental factors like plant genetics and growth stages interact with environmental factors like light, temperature, water, and soil conditions to determine the characteristics and number of secondary metabolites. An understanding of how these factors affect the plant secondary metabolism may be useful in the development of potent herbal medicine (Li et al., 2020).

### 4.1 Gene regulation during abiotic stress in medicinal plants

Omics technologies play a pivotal role in deciphering plant responses to stress at the gene regulatory level. They emphasize the importance of functional genomics, including metabolomics, transcriptomics, and proteomics, in understanding plant molecular responses to stress (Razzaq et al., 2021). The study of epigenomics is considered a valuable method for examining how plants adapt to environmental stresses at the molecular level and how epigenetic information is passed down from one generation to another (Perrone and Martinelli, 2020). Additionally, the role of bioinformatics in analyzing and identifying genetic elements related to stress tolerance is emphasized (Kumar D. et al., 2021). The complex genetic processes underlying stress tolerance and the promise of “omics” technologies in unravelling these mechanisms (Bagati et al., 2018).

Epigenetic modifications, such as histone methylation and DNA methylation, play a crucial role in regulating gene expression in response to abiotic stresses like drought, high salinity, and extreme temperatures (Santos et al., 2011; Shi et al., 2024). These modifications can lead to chromatin remodeling, altering the accessibility of transcription machinery and ultimately affecting plant homeostasis responses (Cadavid et al., 2023). In medicinal plants, these epigenetic changes can influence the production of secondary metabolites, which are responsible for their medicinal properties. Transcription factors, particularly those belonging to families such as WRKY, MYB, AP2/ERF, and NAC, act as mediators in regulating stress responses in plants. NAC transcription factors, for instance, are involved in regulating different signaling pathways of plant hormones that direct a plant’s immunity against pathogens and affect their responses to abiotic stresses. These transcription factors can also influence the biosynthesis of secondary metabolites in medicinal plants, potentially impacting their medicinal properties (Kumar R. et al., 2021).

Researchers have examined how *Polygonatumkingianum*, a medicinal plant in Traditional Chinese Medicine, responds to stress at the gene regulatory level (Qian et al., 2021). The research revealed that drought stress caused specific genes related to various pathways like phenylpropanoid biosynthesis, flavonoid biosynthesis, starch and sucrose metabolism, stilbenoid diarylheptanoid and gingerol biosynthesis, and carotenoid biosynthesis to exhibit different levels of activity an increase in starch and sucrose biosynthesis was evident from transcriptomic changes under drought stress. However, when the plants were rewatered following the period of drought stress, the tubers recovered, and there was an enhanced expression observed in certain genes. Phenylpropanoid and flavonoid biosynthesis pathways were commonly affected across multiple plant species under drought stress. In *Helianthus tuberosus*, genes related to these pathways were differentially expressed (Zhou et al., 2021). Similarly, in *Polygonatumkingianum*, drought stress reduced the expression of genes involved in lignin, gingerol, and flavonoid biosynthesis (Qian et al., 2021). In *Arabidopsis thaliana*, drought conditions led to significant changes in enzymes involved in lignin biosynthesis, such as phenylalanine lyase (PAL) and Caffeoyl Coenzyme A 3-O-methyltransferase 1 (COMT) (Lindberg et al., 2014). *Ligularia fischeri*, drought stress increased the expression of flavone synthase (LfFNS) and anthocyanin 5-O-glucosyltransferase (LfA5GT1), leading to higher levels of flavones and anthocyanins, while decreasing the expression of genes involved in caffeoylquinic acid biosynthesis (Park et al., 2023). Key genes in these pathways, such as phenylalanine ammonia-lyase (PAL), chalcone synthase (CHS), flavonol synthase (FLS), and anthocyanidin synthase (ANS), show increased expression under drought conditions (Ahmed et al., 2021; Park et al., 2023).

## 5 The effects of climate change on medicinal plants

The life cycles of plants are indeed intricately linked with seasonal changes and are susceptible to alterations due to both natural variations and climate change. The phenomenon of global climate change has brought about disruptions in ecosystems and biodiversity, affecting various species. While many plants are impacted, it is noteworthy that only a subset of medicinal plant species is experiencing adverse effects due to these shifts in plant phenology. These changes primarily involve modifications in crucial aspects such as fruit and flower production (Chandra et al., 2020, 2022a), the growth patterns of leaves and buds, and the timing of leaf shedding, especially in autumn or during dry spells (Bidart-Bouzat and Imeh-Nathaniel, 2008).

The rise in global temperatures is expected to significantly affect the synthesis of secondary metabolites in medicinal plants. Metabolites present in medicinal plants, including immune suppressants, anti-diabetic, and anti-cancer agents, play pivotal roles in the plant’s interactions with its environment and have significant implications for human health. These natural compounds are essential for maintaining the delicate balance between plants and their surroundings, while also providing us with crucial remedies to combat various diseases (Sun et al., 2023). Recent research indicates that plants produce secondary metabolites in response to stressful conditions. Furthermore, it is anticipated that the secondary metabolism of plants will be significantly influenced by the major shifts in global climate that are predicted to occur (Pant et al., 2021). Although there is still insufficient research conducted on the impact of climate change-induced temperature rise on plant secondary metabolism (Loreto et al., 2006). The impact of various abiotic factors on medicinal plants has been discussed under the following subheadings.

### 5.1 Impact of elevated CO_2_

**Figure 2:**
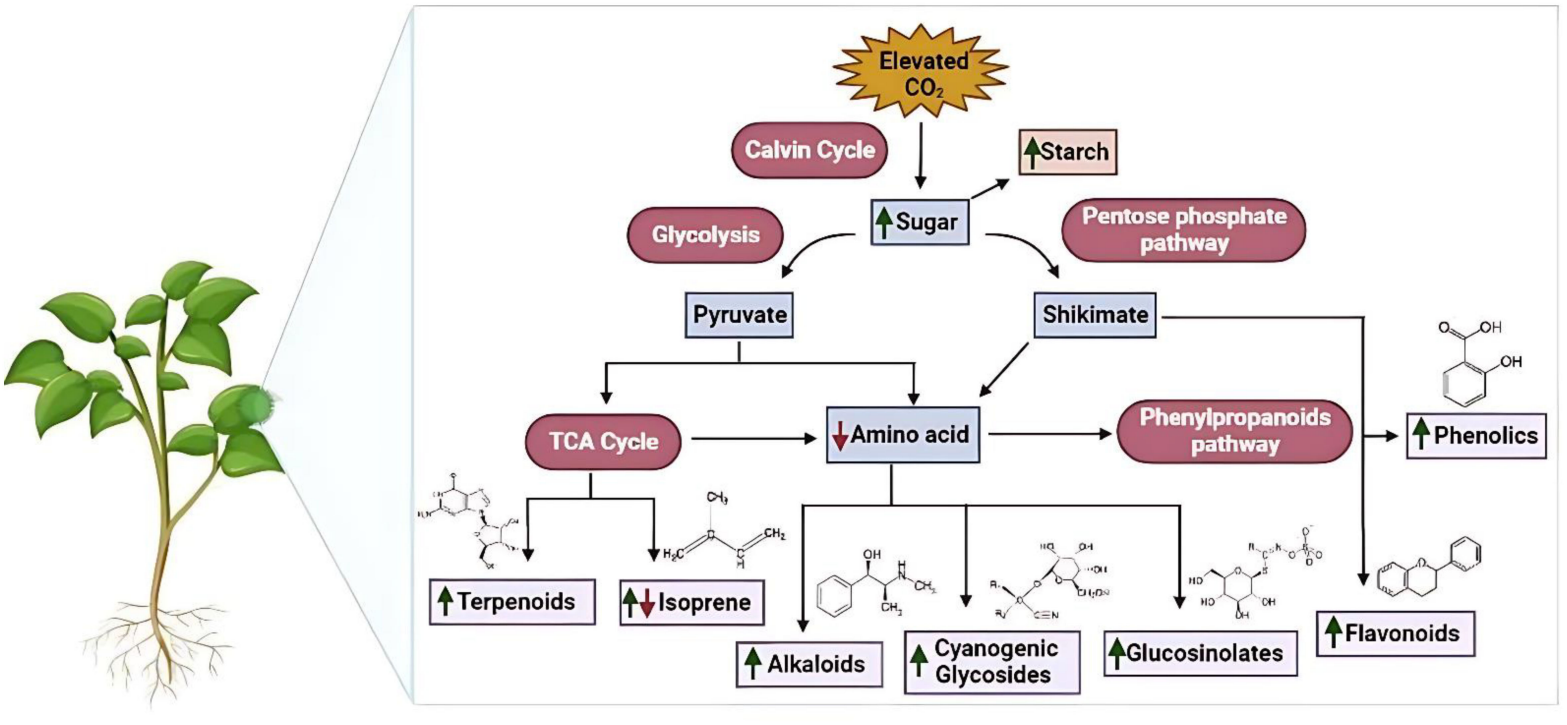
Elevated CO_2_ effects on secondary metabolite synthesis in medicinal plants. The figure highlights metabolic pathways influenced by elevated CO_2_ and associated physiological responses such as enhanced biomass and altered phytochemical concentrations.

The presence of secondary metabolites in medicinal plant species helps them to sustain in adverse climatic conditions. These compounds in plants are secreted through various metabolic pathways (**Figure 2**) and the concentration of these secondary metabolites increases or decreases in environmental stresses to prevent cellular damage. This adaptive behavior affects the therapeutic properties of plants which is due to the concentration of secondary metabolites (Mishra, 2016).The results of experiments conducted under normal climate conditions indicate that the increase of carbon dioxide (CO_2_) has positive effects on various plant parts and products that are utilized in pharmaceuticals. The comparison was made between shoots that were grown in a culture medium with ambient air containing 3000μL CO_2_/L and those that were grown in an elevated CO_2_ environment. The plant species, including *Mentha* sp*icata, Thymus vulgaris, Ocimumbasilicum*, and *Origanum vulgare*, demonstrated encouraging outcomes by displaying a notable rise in both leaf and root counts, accompanied by an increase in overall fresh weight. In other words, these plants responded positively to the treatment, resulting in a significant boost in their growth and overall health (Tisserat and Vaughn, 2001; **Table 1**).

**Table 1:** Impact of elevated CO_2_ on the plant secondary metabolites.

S.no.	Plant Species	Family	Impact	Reference
1	*Valeriana jatamansi*	Caprifoliaceae	Increase in Essential oil (β-patchoulene, patchouli alcohol, germacrene D, bornyl acetate)	(Kaundal et al., 2018)
2	*Paris polyphylla*	Melanthiaceae	Increase in Saponins (Pennogenin and Diosgenin)	(Qiang et al., 2020)
3	*Hibiscus sabdariffa.*	Malvaceae	Increase in total Anthocyanins and Phenolic	(Ali et al., 2019)
4	*Arabidopsis thaliana (L.)*	Brassicaceae	Decrease in Glucosinolates	(Paudel et al., 2016)
5	*Ginkgo biloba*	Ginkgoaceae	Increase in quercetin aglycon (flavonoid), Increase in isorhamnetin and bilobalide and the level of kaempferol aglycon (a type of flavonoid) have decreased.	(Huang et al., 2010)
6	*D. lanata*	Plantaginaceae	Increase in Cardenolide glycoside and Digoxin	(Stuhlfauth et al., 1987)
7	*Heritiera littoralis*	Malvaceae	Increase in Pancratistatin,7-deoxy-trans dihydronarciclasin and 7- deoxynarciclasin (Alkaloids)	(Idso et al., 2000)
8	*Papaver setigerum*	Papaveraceae	Increase in Codeine, Noscapine, Morphine and Papaverine (Alkaloids)	(Ziska et al., 2008)
9	*Mentha piperita*	Lamiaceae	Increase in Flavonoids	(Al Jaouni et al., 2018)
10	*C. roseus*	Apocynaceae	Increase in Alkaloids, Flavonoids Tannins and Phenolic content	(Ezuruike and Prieto, 2014)
11	*Zingiber officinale*	Zingiberaceae	Increase in Phenolic and Flavonoids	(Ghasemzadeh et al., 2010)
12	*Pseudotsugamenziesii*	Pinaceae	Decrease in Terpenes (Monoterpenes)	(Snow et al., 2003)
13	*Q. iliifolia*	Fagaceae	Increase in Phenolic and Tannins	(Ibrahim and Jaafar, 2012)
14	*Stevia rebaudiana*	Asteraceae	Increase in Steviol Glycosides	(Hussin et al., 2017)
15	*Hypericum perforatum*	Hypericaceae	Increase in Phenolics (Hyperforin, Hypericin and Pseudohypericin)	(Zobayed and Saxena, 2004)

The concentration of secondary metabolites in plants is influenced not only by the concentration of CO_2_ but also by the duration of exposure. For instance, the bulbs of the beach spider lily, scientifically known as *Hymenocallis littoralis*, are famous for their ability to combat cancer and viruses. In a study conducted on these bulbs, the concentrations of three alkaloids, namely pancratistatin, 7-deoxy-trans dihydronarciclasin, and 7-deoxynarciclasine, were found to increase steadily during the first year of the experiment, but after that period, there was a notable reduction in their level of concentration (Idso et al., 2000).

### 5.2 Effect of elevated ozone

The presence of ozone in the environment can impact the synthesis of secondary compounds in plants (**Table 2**). Elevated levels of ozone (O_3_) have been shown to activate important metabolic pathways, including the jasmonic acid and salicylic acid pathways, which are essential for the synthesis of secondary metabolites in plants. These pathways are typically triggered in response to physiological stress. When plants are exposed to increased levels of ozone, these metabolic pathways can be activated as a natural response to the stress induced by elevated ozone levels. This activation stimulates the generation of secondary metabolites, helping plants cope with the environmental challenge without compromising their overall health and survival (Mishra, 2016).

**Table 2:** Impact of elevated ozone (O_3_) on the Plant secondary metabolites.

S.no.	Plant Species	Family	Impact	Reference
1	*Betula pendula*	Betulaceae	Increase in a-flavonoid and hyperoside. Decrease in Phenolics (papyriferic acid, betuloside, dehydrosalidroside and hyperoside)	(Lavola et al., 1994)
2	*Salvia officinalis*	Lamiaceae	Increase in Caffeic acid, Gallic acid, Catechinic acid and Rosmarinic acid (Phenolic)	(Pellegrini et al., 2015)
3	*Pueraria* *thomsonii*	Fabaceae	Increase in Isoflavones	(Sun et al., 2012)
4	*Pinus taeda*	Pinaceae	Increase in Tannins	(Jordan et al., 1991)
5	*Melissa officinalis*	Lamiaceae	Decrease in Dihydrocapsaicin and Capsaicin	(Shakeri et al., 2016)
6	*C. baccatum*	Solanaceae	Increase in Phenolics, Tannins and Anthocyanins.	(Bortolin et al., 2016)
7	*Hypericum perforatum*	Hypericaceae	Increase in Flavonoid and Phenols	(Pellegrini et al., 2018)

### 5.3 Impact of ultraviolet-B radiation

UV-B radiation has the potential to inflict harm on a range of biomolecules, including DNA, proteins, and other essential cellular constituents. The exposure to this form of radiation holds the capacity to influence the overall growth and developmental processes of plants, resulting in alterations to their reproductive and vegetative biomass, height, flowering schedules and leaf characteristics (Mishra, 2016; **Table 3**).

**Table 3:** Impact of UV-B radiation on secondary metabolites of plants.

S.no.	Plant Species	Family	Impact	Reference
1	*Withaniasomnifera*	Solanaceae	Increase in Flavonoids, Tannins and Anthocyanins	(Takshak and Agrawal, 2014).
2	*Curcuma longa*	Zingiberaceae	Decrease in Phenols, PAL enzyme and Peroxidase	(Ferreira et al., 2016).
3	*Chrysanthemum morifolium*	Asteraceae	Increase in Phenols (Phenolic Acid)	(Ma et al., 2016)
4	*Coleus forskohlii*	Lamiaceae	Increase in Flavonoids and Phenolics	(Takshak and Agrawal, 2015)
5	*Prunella vulgaris*	Lamiaceae	Increase in flavonoids, rosmarinic acid, caffeic acid	(Chen et al., 2018)
6	*Kalanchoe pinnata* (Lam.)	Crassulaceae	Increase in Phenolic and Flavonoid	(dos Santos Nascimento et al., 2015)
7	*Nasturtium officinale*	Brassicaceae	Increase in Glucosinolate	(Reifenrath and Müller, 2007)
8	*Arnica montana*	Asteraceae	Increase in Lignin, Anthocyanins, Phenolic acids and Tannins	(Spitaler et al., 2006)
9	*Astragalus compactus*	Fabaceae	Increase in Tannins, Lignin, Phenolic acids and Anthocyanins	(Naghiloo et al., 2012)
10	*Asparagus officinalis*	Asparagaceae	Increase in Flavonol	(Eichholz et al., 2012)

When reactive oxygen species (ROS) are generated, they initiate a defensive signaling pathway, stimulating the synthesis of secondary metabolites. These metabolites can absorb Ultraviolet-B radiation and include compounds such as anthocyanins, alkaloids, flavonoids, lignin, tannins, phytosterols and saponins (Takshak and Agrawal, 2014; dos Santos Nascimento et al., 2015). Furthermore, changes in the functioning of antioxidant enzymes can be noted, as demonstrated by enzymes originating from the phenylpropanoid pathway (Takshak and Agrawal, 2014). Anthocyanins, a crucial class of pigments discovered in fruits, flowers, and leaves, are predominantly located within the epidermal cells of flowers and other plant components. They play a regulatory role in absorbing ultraviolet-B radiation. Phenolic compounds are abundantly found in plants, and numerous investigations have substantiated their augmentation in enzymatic activities across diverse studies. During exposure to ultraviolet-B stress, flavonoids are produced through enzymatic processes within the phenylpropanoid pathway. Exposure to ultraviolet-B radiation enhances the activation of specific genes responsible for encoding enzymes directly engaged in anthocyanin biosynthesis. Increased enzyme activities, such as Chalcone synthase (CHS), Flavanone 3-hydroxylase (F3H), and Dihydroflavonol reductase (DFR), enhance the anthocyanin content within the plant (Park et al., 2007).

### 5.4 Impact of temperature on secondary metabolites

A suitable temperature is necessary for plant development. Plants that experience heat or cold stress are negatively impacted by high and low temperatures, respectively (Yadav, 2010).

#### 5.4.1 Impact of heat stress

At temperatures higher than optimal, plants experience heat stress. It affects the stomatal conductance, which in turn slows down photosynthesis and plant development. The synthesis of PSMs is also affected by high temperatures. The photosystem II exhibits a decrease in photochemical effectiveness at high temperatures, which increases plant stress. Several research studies have provided evidence indicating that elevated temperatures can trigger the increased production of secondary metabolites (**Table 4**). However, contrasting findings have also been observed in certain studies, where secondary metabolite levels showed a decline. For instance, in the case of *Panax quinquefolius* plants, an increase in temperature was found to result in an augmentation of root ginsenoside quantities and in another study on two high-altitude aromatic plants viz., *Angelica glauca* and *Nardostachysjatamansi*, monoterpenes were found to be decreased in response to elevated CO_2_ and temperature gradients (Jochum et al., 2007; Dobhal et al., 2024).

**Table 4:** Impact of Heat stress on the secondary metabolites of plants.

S.no.	Plant Species	Family	Impact	Reference
1	*Salvia miltiorrhiza*	Lamiaceae	Increase in Tanshinone	(Zhang et al., 2019)
2	*Tithonia diversifolia*	Asteraceae	Increase in Phenols	(Sampaio et al., 2016)
3	*P. minus*	Polygonaceae	Increase in Flavonols (Favonoid)	(Goh et al., 2015)
4	*Duboisiamyoporoides*	Solanaceae	Increase in Alkaloids	(Ullrich et al., 2017)
5	*Dendrobium officinale*	Amaranthaceae	Decrease in Alkaloids and Flavonoid	(Yuan et al., 2020)
6	*Ocimum* *basilicum*	Lamiaceae	Increase in Salicylic acid and enhance plant tolerance	(Clarke et al., 2004)
7	*A. sinensis*	Thymelaeaceae	Increase in Jasmonic Acid	(Xu et al., 2016)
8	*Silybum marianum*	Asteraceae	Decrease in Silymarin	(Rahimi and Hasanloo, 2016)
9	*Perilla frutescens*	Lamiaceae	Decrease in Anthocyanins	(Zhong and Yoshida, 1993)
10	*Panax* *quinquefolius*	Araliaceae	Increase in Saponins (Ginsenoside) anddecrease in photosynthesis rate	(Jochum et al., 2007)
11	*Eleutherococcussenticosus*	Araliaceae	Increase in Chlorogenic acid and Eleutherosides	(Shohael et al., 2006)
12	*Astragalus compactus*	Fabaceae	Increase in Phenolics	(Naghiloo et al., 2012)
13	*Hypericum perforatum*	Hypericaceae	Decrease in Hyperforin and Increase in Naphthodianthrones and phenolics	(Radušienė et al., 2012)
14	*Chrysanthemum* spp.	Asteraceae	Decrease in Jasmonic acid, α-linolenic and Anthocyanins	(Shibata et al., 1988)

Heat stress induces changes in gene expression that facilitate the activation of molecular mechanisms protecting plants against heat stress (Zhu et al., 2013). A majority of these genes control the production of regulatory, transporter, detoxifying, and Osmo-protective proteins. When a plant is exposed to high temperatures, it can develop an ability to tolerate heat through either adaptation or acclimation. This is achieved by changing its biochemical and physiological processes, which are influenced by modifications in gene expression (Mirza et al., 2010).

#### 5.4.2 Impact of cold stress

Plants are adversely affected by low temperatures, which can result in stress and various negative consequences such as decreased growth, reduced productivity, loss of diversity, and limited distribution (Chinnusamy et al., 2007; Rahman, 2013) (**Table 5**). Plants can alter their coping mechanisms to tolerate cold stress by transferring resources and slowing development (Eremina et al., 2016). Plant physiology is directly impacted by low temperatures (Ruelland et al., 2009). The concentration of cellular membrane fluidity is altered by low temperatures (Upchurch, 2008; Sevillano et al., 2009). Plants possess the capacity to undergo physiological, biochemical, and molecular alterations in response to low temperatures, a phenomenon commonly referred to as cold acclimation. This adaptive mechanism enables them to better tolerate and survive in colder environments. When plants are exposed to cold stress, they frequently show a reduction in chlorophyll a levels and overall chlorophyll content, accompanied by an increase in leaf apoplastic and total soluble protein levels (Zhou et al., 2021; Aazami et al., 2021). These adaptations serve as strategies employed by plants to effectively adapt to and endure the adverse effects of low temperatures. By modifying these sentences, the phrasing and structure have been altered to reduce plagiarism and paraphrase the information (Esra et al., 2010). When plants are exposed to low temperatures, the production of free oxygen radicals increases, leading to elevated internal stress levels in plant cells. In response, plants activate antioxidants to counteract and eliminate these radicals (Sevillano et al., 2009 & Ruelland et al., 2009).To endure unfavorable circumstances, plant cells employ strategies to uphold their structural integrity, including augmenting their amino acid content, soluble solids, and cryoprotective proteins. This process involves the activation of different enzymatic and metabolic pathways (Ruelland et al., 2009; Eremina et al., 2016).

**Table 5:** Impact of Cold stress on the secondary metabolites of plants.

S.no.	Plant Species	Family	Impact	Reference
1	*Teucrium polium*	Lamiaceae	Increase in Phenols, Flavonoids and Essential oils and Decrease in Idoids and Diterpenes	(Lianopoulou et al., 2014)
2	*Withania* *somnifera*	Solanaceae	Increase in Alkaloids (steroidal lactone withanolides)	(Mir et al., 2015)
3	*Salvia sclarea*	Lamiaceae	Increase in Essential oils	(Kaur et al., 2015)
4	*A. thaliana*	Brassicaceae	Increase in sterol glycosides	(Mishra et al., 2013)
5	*Ocimumtenuiflorum*	Lamiaceae	Decrease in Eugenol and methyleugenol contents	(Rastogi et al., 2019)
6	*Camellia japonica*	Theaceae	Increase in Jasmonic acid and α-linolenic acid (ALA)	(Li et al., 2016)
7	*Artemisia annua*	Asteraceae	Increase in Artemisinin	(Zeng et al., 2008)
8	*Vitis vinifera*	Vitaceae	Decrease in p-Coumaric acid, Caffeic acid and Ferulic acid	(Król et al., 2015)
9	*Cucumis sativus*	Cucurbitaceae	Increase in Flavonoids, Lignin, Phenols, Cinnamic acid, ρ - Coumaric acid, Ferulic acid and Caffeic acid	(Chen et al., 2013)
10	*A. tilesii*	Asteraceae	Decrease in Flavonoids	(Havryliuk et al., 2017)

Certain medicinal plants, including *Teucrium polium*, *Thymus sibthorpii, Phlomisfruticosa, Saturejathymbra*, and *Cistus incanus*, exhibit a phenomenon known as seasonal dimorphism. This characteristic enables them to employ specific defense strategies that vary depending on the particular season (Lianopoulou et al., 2014). *Origanum dictamnus* plants exhibit seasonal variation through different defense mechanisms triggered by various hormones. To cope with cold temperatures, these plants undergo several adaptations such as altering leaf size, shape, and arrangement. They also develop a waxy coating, thicker cuticles, and a dense layer of non-glandular hairs on the leaf epidermis. These modifications aid in cold tolerance. Similarly, in high temperatures, the plants’ mesophyll cells possess enlarged intracellular spaces to efficiently store air (Lianopoulou and Bosabalidis, 2014). The essential oils in these plants seem to vary in composition depending on external temperature changes, as evidenced by the fact that during winter, p-Cymene concentrations were at 60%, while during summer, carvacrol concentrations were at 42% (Lianopoulou and Bosabalidis, 2014).

### 5.5 Impact of drought stress

Drought occurs when plants are unable to obtain an adequate water supply, which leads to a decline in turgor and water potential. This water deficiency hampers their normal physiological processes, resulting in disruptions to their regular functions (Lisar et al., 2012).When stomata close, the photosynthetic rate decreases, the activity of ATP synthesis enzymes diminishes, and cell membranes can suffer damage. Furthermore, osmotic stress caused by drought negatively impacts the production of cereal crops (Valentovic et al., 2006).

Drought stress during the cultivation of spices and medicinal plants can influence the contents of secondary metabolites. The studies found that plants tend to accumulate increased levels of specific natural compounds, including isoprenoids, alkaloids, flavones and phenols under conditions of drought stress (**Table 6**). This increase in secondary metabolites is attributed to metabolic reactions triggered by a lack of water, which influences the rate of metabolic activities in plants. It is crucial to emphasize that extended periods of drought can result in decreased levels of secondary metabolites in plants. This decline can be attributed to significant reductions in overall plant growth during prolonged drought phases. The application of stress signal transducers, like salicylic acid and methyl jasmonate, can also enhance the concentrations of specific bioactive compounds. Overall, the findings suggest that intentionally causing a significant amount of drought stress while cultivating can enhance the quality and quantity of secondary metabolites in medicinal plants (Gabbish et al., 2015; Shil and Dewanjee, 2022; Kleinwächter and Selmar, 2015).

**Table 6:** Impact of Drought stress on the plant secondary metabolites.

S.no.	Plant Species	Family	Impact	Reference
1	*Trachyspermumammi*	Apiaceae	Increase in Phenolic content	(Azhar et al., 2011).
2	*Matricaria chamomilla*	Asteraceae	Decrease in Essential oil content	(Razmjoo et al., 2008).
3	*Ocimum americanum*	Lamiaceae	Increase in essential oil and Decrease in N, P, K and protein content.	(Khalid, 2006).
4	*Artemisia annua*	Asteraceae	Increase in Artemisinin	(Zobayed et al., 2007).
5	*Glechoma longituba*	Lamiaceae	Decrease in Flavonoids	(Zhang et al., 2012).
6	*Petroselinum* *crispum*	Apiaceae	Increase in Monoterpenes	(Petropoulos et al., 2008)
7	*Labisia pumila*	Primulaceae	Increase in Flavonoids and Phenolics	(Jaafar et al., 2012)
8	*S. officinalis*	Lamiaceae	Increase in Monoterpenes	(Nowak et al., 2010)
9	*Hypericum* *brasiliense*	Hypericaceae	Increase in Rutin, Quercetin, Betulinic acid and phenolic contents	(Verma and Shukla, 2015)
10	*Scrophularianingpoensis*	Scrophulariaceae	Increase in Glycosides	(Wang et al., 2010)
11	*Papaver somniferum*	Papaveraceae	Increase in Morphine Alkaloids	(Szabó et al., 2003)
12	*B. napus*	Brassicaceae	Increase in Glucosinolates	(Jensen et al., 1996)
13	*Salvia miltiorrhiza*	Lamiaceae	Increase in Rosmarinic acid	(Liu et al., 2011)
14	*O. basilicum*	Lamiaceae	Increase in essential oil and Decrease in N, P, K and protein content.	(Khalid, 2006).
15	*Hypericum brasiliense*	Hypericaceae	Increase in Rutine,Quercetin and Betulinic acid	(de Abreu and Mazzafera, 2005)
16	*Thymus vulgaris*	Lamiaceae	Increase in p-cymene and carvacrol,γ-terpinene and Decrease in Thymol	(Alavi-Samani et al., 2015) & (Mohammadi et al., 2018)
17	*Scutellariabaicalensis*	Lamiaceae.	Increase in Baicalin	(Cheng et al., 2018)

A number of previous research studies have explored the impact of drought conditions on the synthesis of bioactive compounds in plants. The findings consistently indicate that in response to drought stress, plants augment the production of secondary metabolites, like terpenoids, alkaloids, phenolics, glucosinolates, and cyanogenic glucosides. Concurrently, these plants experience a reduction in their growth rate due to the constraints imposed by limited water availability (Yeshi et al., 2022). When the production of biomass is diminished, it typically leads to an elevation in the levels of secondary metabolites within plants. However, this increase in concentration is not due to a faster rate of metabolite synthesis but rather is dependent on whether the weight of the plant material is measured in terms of fresh or dry weight. In other words, whether the total concentration of secondary metabolites increases or not depends on the method used to measure the weight of the plant material (Kleinwächter and Selmar, 2015).

In a study by (Nowak et al., 2010) on *Salvia officinalis*, the monoterpene concentration increased dramatically under water stress;this elevation in monoterpene concentration was considerably greater in contrast to the decrease in biomass observed in the control group, which was grown under conditions of adequate water supply. *Petroselinum crispum*, also known as parsley, was the focus of an experiment that showed increases in monoterpene concentration to be substantially greater than decreases in leaf biomass (Petropoulos et al., 2008). *Origanum vulgare* had stable essential oil content per plant, but when it was under drought conditions, the amount of metabolites increased (Ninou et al., 2017). According to (Paulsen and Selmar, 2016), the production of monoterpenes remained consistent despite a reduction in the amount of biomass used. They explained the increase in monoterpene content in thyme plants based on this observation. Additionally, the concentration of n-monoterpenes did not change overall.

Dry weight measurements revealed that the rate of synthesis differed between drought-stressed plants and control plants cultivated under optimal watering conditions. Specifically, in the early stages of the experiment, stressed plants showed significantly higher rates of synthesis than the control group, as measured by dry weight. However, this finding changed when the duration of drought was extended (Paulsen and Selmar, 2016). A similar trend was seen in the levels of phenolic compounds in *Hypericum brasiliense* during periods of drought. Under the specified conditions, there was a noteworthy rise in both the quantity and potency of these compounds. This escalation in the phenolic content resulted in reduced plant sizes in stressed specimens as opposed to those grown under normal conditions (de Abreu and Mazzafera, 2005).

### 5.6 Impact of salinity

Exposing cells to a high concentration of salt causes them to lose water from their cytoplasm, which creates osmotic pressure. High salt concentrations in plants can induce ionic and osmotic stresses, leading to a decrease in cytosol and vacuole volumes. This exposure can also trigger alterations in different secondary metabolite concentrations (Mahajan and Tuteja, 2005) (**Table 7**). According to reports, there is evidence to suggest that under conditions of salt stress, there is an observed elevation in the concentrations of anthocyanins (Parida and Das, 2005). Certain species were found to be sensitive to salt stress, and as a result, they showed a reduction in the level of anthocyanin compared to other species that were not affected by salt stress and had no change in the quantity of anthocyanin present (Daneshmand et al., 2010). Plants respond to increased salinity by activating enzymes and regulatory genes that affect the production of secondary metabolites. The quantity of these metabolites that the plant produces changes depending on its particular requirements (Punetha et al., 2022).

**Table 7:** Impact of Salt stress on the secondary metabolites of plants.

S.no.	Plant Species	Family	Impact	Reference
1	*Rauvolfiatetraphylla*	Apocynaceae	Increase in Reserpine	(Said-Al Ahl and Omer, 2011).
2	*R. communis*	Euphorbiaceae	The concentration of ricinine alkaloid is higher in the plant’s aerial portions, while it is lower in the underground portions.	(Said-Al Ahl and Omer, 2011).
3	*Solanum nigrum*	Solanaceae	Increase in alkaloids	(Said-Al Ahl and Omer, 2011)
4	*Nigella sativa*	Ranunculaceae	Enhancement of phenols content	(Bourgou et al., 2010)
5	*Satureja hortensis*	Lamiaceae	Decrease in essential oil content	(Said-Al Ahl and Omer, 2011).
6	*Mentha piperita*	Lamiaceae	Alter the concentration of menthol and rosmarinic acid	(Tabatabaie and Nazari, 2007)
7	*Mentha pulegium*	Lamiaceae	Enhancement of phenols content	(Oueslati et al., 2010)
8	*C. roseus*	Apocynaceae	Decrease in protein content and increase in vincristine alkaloids	(Osman et al., 2007)
9	*Matricaria* *chamomilla*	Asteraceae	Increase in Phenolic content (Protocatechuic, Chlorogenic and Caffeic acids)	(Kováčik et al., 2009)
10	*Satureja hortensis*	Lamiaceae	Increase in Flavonoids and Phenolic	(Najafi and Khavari-Nejad, 2010)
11	*Datura innoxia*	Solanaceae	Increase in Tropane alkaloids (TAs)	(Brachet and Cosson, 1986)
12	*Cakile maritima*	Brassicaceae	Increase in Polyphenol	(Ksouri et al., 2007)
13	*Trifolium repens*	Fabaceae	Increase in Glycinebetaine	(Varshney et al., 1988)
14	*Matricaria recutita*	Asteraceae	Decrease in essential oil content	(Said-Al Ahl and Omer, 2011).
15	*Plantago ovata*	Plantaginaceae	Increase in Flavonoids Saponins and Proline	(Haghighi et al., 2012).
16	*Achillea fragratissima*	Asteracaea	Increase in both Alkaloid and Tannin	(Abd EL-Azim and Ahmed, 2009)
17	*C. sativum*	Apiaceae	Increase in Carvacrol (Monoterpenoid phenol), octanaldehyde and Borneol (Terpene alcohol) and decrease in α-Pinene, γ-trepineandMyroxide	(Neffati and Marzouk, 2008)
18	*Origanum majorana*	Lamiaceae	Increase in Linalyl acetate and Terpinene-4-ol	(Baatour et al., 2010)

## 6 Adaptation with climate change and global warming

Medicinal plants are facing significant challenges due to climate change and global warming, necessitating adaptation strategies to ensure their survival and continued availability for human use. Climate change is affecting the distribution, growth, and chemical composition of medicinal plants. Rising temperatures and changing precipitation patterns are altering suitable habitats for these species, potentially leading to population declines (Cahyaningsih et al., 2021). Additionally, environmental stresses can impact the production of secondary metabolites, which affect the therapeutic potential of medicinal plants (Harish et al., 2012). The key adaptation strategies may include conserving threatened species (both *in-situ* and *ex-situ*), promoting local cultivation, training harvesters in sustainable practices, certifying commercial material, and monitoring raw material quality to ensure efficacy (Applequist et al., 2020; Cahyaningsih et al., 2021).

## 7 Conclusion and future recommendations

The production and variation of secondary metabolites play a significant role in enabling plants to adapt and thrive in diverse environmental conditions. Indigenous plant species are particularly vulnerable to climate change, which can affect their secondary metabolite production, threatening their survival. The environmental stressors like elevated carbon dioxide levels, temperature stress, drought, and high salinity can enhance the production of secondary metabolites. However, these environmental factors may also negatively affect plant growth and overall productivity. The development of effective and sustainable strategies to enhance secondary metabolite synthesis in plants facing climate change requires future research on the molecular mechanisms controlling biosynthesis and the combined effects of multiple environmental factors on their production.

## References

[B1] Aazami M. A. Asghari-Aruq M. Hassanpouraghdam M. B. Ercisli S. Baron M. Sochor J. (2021). Low temperature stress mediates the antioxidants pool and chlorophyll fluorescence in *Vitis vinifera* L. cultivars. Plants 10, 1877. doi: 10.3390/plants10091877 34579411 PMC8470009

[B2] Abd EL-Azim W. M. Ahmed S. T. (2009). Effect of salinity and cutting date on growth and chemical constituents of Achillea fragratissimaForssk, under Ras Sudr conditions. Res. J. Agr. Biol. Sci. 5, 1121–1129.

[B3] Ahmed U. Rao M. J. Qi C. Xie Q. Noushahi H. A. Yaseen M.. (2021). Expression profiling of flavonoid biosynthesis genes and secondary metabolites accumulation in populus under drought stress. Molecules 26, 5546. doi: 10.3390/molecules26185546 34577017 PMC8467073

[B4] Akula R. Ravishankar G. A. (2011). Influence of abiotic stress signals on secondary metabolites in plants. Plant Signal. Behav. 6, 1720–1731. doi: 10.4161/psb.6.11.17613 22041989 PMC3329344

[B5] Alavi-Samani S. M. Kachouei M. A. Pirbalouti A. G. (2015). Growth, yield, chemical composition, and antioxidant activity of essential oils from two thyme species under foliar application of jasmonic acid and water deficit conditions. Hortic. Environ. Biotechnol. 56, 411–420. doi: 10.1007/s13580-015-0117-y

[B6] Ali S. A. M. Latip J. Zain C. R. C. M. (2019). “Growth and phenolic constituents production in roselle (Hibiscus sabdariffa var. UKMR-2) as influenced by irrigation treatment,” in AIP Conference Proceedings, Vol. 2111. (AIP Publishing) (1). doi: 10.1063/1.5111261

[B7] Al Jaouni S. Saleh A. M. Wadaan M. A. Hozzein W. N. Selim S. AbdElgawad H. (2018). Elevated CO2 induces a global metabolic change in basil (Ocimumbasilicum L.) and peppermint (Mentha piperita L.) and improves their biological activity. J. Plant Physiol. 224, 121–131. doi: 10.1016/j.jplph.2018.03.016 29626813

[B8] Applequist W. L. Brinckmann J. A. Cunningham A. B. Hart R. E. Heinrich M. Katerere D. R.. (2020). Scientists’ warning on climate change and medicinal plants. Planta Med. 86, 10–18. doi: 10.1055/a-1113-1659 31731314

[B9] Azhar N. Hussain B. Ashraf M. Y. Abbasi K. Y. (2011). Water stress mediated changes in growth, physiology and secondary metabolites of desi ajwain (Trachyspermumammi L.). Pakistan J. Bot. 43, 15–19.

[B10] Baatour O. Kaddour R. Aidi Wannes W. Lachaal M. Marzouk B. (2010). Salt effects on the growth, mineral nutrition, essential oil yield and composition of marjoram (Origanum majorana). Acta Physiol. Plant. 32, 45–51. doi: 10.1007/s11738-009-0374-4

[B11] Bagati S. Nazir M. Dar A. A. Zargar S. M. Mahajan R. (2018). “Omics”: a gateway towards abiotic stress tolerance (Springer Singapore), pp. 1–45. doi: 10.1007/978-981-10-7479-0_1

[B12] Bartram S. Jux A. Gleixner G. Boland W. (2006). Dynamic pathway allocation in early terpenoid biosynthesis of stress-induced lima bean leaves. Phytochemistry 67, 1661–1672. doi: 10.1016/j.phytochem.2006.02.004 16580034

[B13] Bidart-Bouzat M. G. Imeh-Nathaniel A. (2008). Global change effects on plant chemical defenses against insect herbivores. J. Integr. Plant Biol. 50, 1339–1354. doi: 10.1111/j.1744-7909.2008.00751.x 19017122

[B14] Bortolin R. C. Caregnato F. F. Junior A. M. D. Zanotto-Filho A. Moresco K. S. de Oliveira Rios A.. (2016). Chronic ozone exposure alters the secondary metabolite profile, antioxidant potential, anti-inflammatory property, and quality of red pepper fruit from Capsicum baccatum. Ecotoxicol. Environ. Saf. 129, 16–24. doi: 10.1016/j.ecoenv.2016.03.004 26970882

[B15] Bourgou S. Kchouk M. E. Bellila A. Marzouk B. (2010). Effect of salinity on phenolic composition and biological activity of Nigella sativa. Acta Hortic. 853, 57–60. doi: 10.17660/actahortic.2010.853.5

[B16] Brachet J. Cosson L. (1986). Changes in the total alkaloid content of Datura innoxia Mill. subjected to salt stress. J. Exp. Bot. 37, 650–656. doi: 10.1093/jxb/37.5.650

[B17] Cadavid I. C. Balbinott N. Margis R. (2023). Beyond transcription factors: more regulatory layers affecting soybean gene expression under abiotic stress. Genet. Mol. Biol. 46, e20220166. doi: 10.1590/1678-4685-gmb-2022-0166 36706026 PMC9881580

[B18] Cahyaningsih R. Phillips J. Brehm J. M. Gaisberger H. Maxted N. (2021). Climate change impact on medicinal plants in Indonesia. Global Ecol. Conserv. 30, e01752. doi: 10.1016/j.gecco.2021.e01752

[B19] Chandra S. Chandola V. Nautiyal M. C. Purohit V. K. (2020). Elevated CO 2 causes earlier flowering in an alpine medicinal herb *Aconitum heterophyllum Wall* . Curr. Sci. 00113891), 118(11).

[B20] Chandra S. Chandola V. Sultan Z. Singh C. P. Purohit V. K. Nautiyal B. P.. (2022b). Climate change adversely affects the medicinal value of *Aconitum* species in Alpine region of Indian Himalaya. Ind. Crops Prod. 186, 115277. doi: 10.1016/j.indcrop.2022.115277

[B21] Chandra S. Singh A. Mathew J. R. Singh C. P. Pandya M. R. Bhattacharya B. K.. (2022a). Phenocam observed flowering anomaly of *Rhododendron arboreum Sm.* in Himalaya: a climate change impact perspective. Environ. Monit. Assess. 194, 877. doi: 10.1007/s10661-022-10466-1 36229620

[B22] Chen S. Jin W. Liu A. Zhang S. Liu D. Wang F.. (2013). Arbuscular mycorrhizal fungi (AMF) increase growth and secondary metabolism in cucumber subjected to low temperature stress. Scientia Hortic. 160, 222–229. doi: 10.1016/j.scienta.2013.05.039

[B23] Chen Y. Zhang X. Guo Q. Liu L. Li C. Cao L.. (2018). Effects of UV-B radiation on the content of bioactive components and the antioxidant activity of Prunella vulgaris L. Spica during development. Molecules 23, 989. doi: 10.3390/molecules23050989 29695057 PMC6099561

[B24] Cheng L. Han M. Yang L. M. Li Y. Sun Z. Zhang T. (2018). Changes in the physiological characteristics and baicalin biosynthesis metabolism of Scutellariabaicalensis Georgi under drought stress. Ind. Crops Prod. 122, 473–482. doi: 10.1016/j.indcrop.2018.06.030

[B25] Chinnusamy V. Zhu J. Zhu J. K. (2007). Cold stress regulation of gene expression in plants. Trends Plant Sci. 12, 444–451. doi: 10.1016/j.tplants.2007.07.002 17855156

[B26] Clarke S. M. Mur L. A. Wood J. E. Scott I. M. (2004). Salicylic acid dependent signalling promotes basal thermotolerance but is not essential for acquired thermotolerance in *Arabidopsis thaliana* . Plant J. 38, 432–447. doi: 10.1111/j.1365-313X.2004.02054.x 15086804

[B27] Daneshmand F. Arvin M. J. Kalantari K. M. (2010). Physiological responses to NaCl stress in three wild species of potato in *vitro* . Acta Physiol. Plant. 32, 91–101. doi: 10.1007/s11738-009-0384-2

[B28] de Abreu I. N. Mazzafera P. (2005). Effect of water and temperature stress on the content of active constituents of Hypericum brasiliense Choisy. Plant Physiol. Biochem. 43, 241–248. doi: 10.1016/j.plaphy.2005.01.020 15854832

[B29] Dobhal P. Purohit V. K. Chandra S. Rawat S. Prasad P. Bhandari U.. (2024). Climate-induced changes in essential oil production and terpene composition in Alpine aromatic plants. Plant Stress 12, 100445. doi: 10.1016/j.stress.2024.100445

[B30] dos Santos Nascimento L. B. Leal-Costa M. V. Menezes E. A. Lopes V. R. Muzitano M. F. Costa S. S.. (2015). Ultraviolet-B radiation effects on phenolic profile and flavonoid content of *Kalanchoe pinnata* . J. Photochem. Photobiol. B: Biol. 148, 73–81. doi: 10.1016/j.jphotobiol.2015.03.011 25900552

[B31] Eichholz I. Rohn S. Gamm A. Beesk N. Herppich W. B. Kroh L. W.. (2012). UV-B-mediated flavonoid synthesis in white asparagus (Asparagus officinalis L.). Food Res. Int. 48, 196–201. doi: 10.1016/j.foodres.2012.03.008

[B32] Eremina M. Rozhon W. Poppenberger B. (2016). Hormonal control of cold stress responses in plants. Cell. Mol. Life Sci. 73, 797–810. doi: 10.1007/s00018-015-2089-6 26598281 PMC11108489

[B33] Esra K.O. Ç. İŞLEK C. Üstün A. S. (2010). Effect of cold on protein, proline, phenolic compounds and chlorophyll content of two pepper (*Capsicum annuum L.*) varieties. Gazi Univ. J. Sci. 23, 1–6.

[B34] Ezuruike U. F. Prieto J. M. (2014). The use of plants in the traditional management of diabetes in Nigeria: pharmacological and toxicological considerations. J. Ethnopharmacol. 155, 857–924. doi: 10.1016/j.jep.2014.05.055 24929108

[B35] Ferreira M. I. Uliana M. R. Costa S. M. Magro M. Vianello F. Ming L. C.. (2016). Exclusion of solar UV radiation increases the yield of curcuminoid in Curcuma longa L. Ind. Crops Prod. 89, 188–194. doi: 10.1016/j.indcrop.2016.05.009

[B36] Gabbish A. A. Klenwachter M. Selmar D. (2015). Influencing the content of secondary metabolites in spice and medicinal plants by deliberately applying drought stress during their cultivation. Jordan J. Biol. Sci. 8, 1–10.

[B37] Ghasemzadeh A. Jaafar H. Z. Rahmat A. (2010). Elevated carbon dioxide increases contents of flavonoids and phenolic compounds, and antioxidant activities in Malaysian young ginger (Zingiber officinale Roscoe.) varieties. Molecules 15, 7907–7922. doi: 10.3390/molecules15117907 21060298 PMC6259178

[B38] Goh H. ‐H Sukiran N. A. Baharum S. N. Khairudin K. Normah M. N. (2015). Metabolite profiling reveals temperature effects on the VOCs and flavonoids of different plant populations. Plant Biology, 18(S1), 130–139. doi: 10.1111/plb.12403 26417881

[B39] Guerriero G. Berni R. Muñoz-Sanchez J. A. Apone F. Abdel-Salam E. M. Qahtan A. A.. (2018). Production of plant secondary metabolites: Examples, tips and suggestions for biotechnologists. Genes 9, 309. doi: 10.3390/genes9060309 29925808 PMC6027220

[B40] Haghighi Z. Karimi N. Modarresi M. Mollayi S. (2012). Enhancement of compatible solute and secondary metabolites production in Plantago ovata Forsk. by salinity stress. J. Medicinal Plants Res. 6, 3495–3500.

[B41] Harish B. S. Dandin S. B. Umesha K. Sasanur A. (2012). Impact of climate change on medicinal plants-A review. Anc Sci. Life 32, S23.

[B42] Havryliuk O. Мatvieieva N. Tashyrev O. Yastremskaya L. (2017). Influence of cold stress on growth and flavonoids accumulation in *Artemisia tilesii* “hairy” root culture. Agrobiodiversity Improv. Nutr. Health Life Qual. (1), 163–167.

[B43] Hu L. Robert C. A. Cadot S. Zhang X. I. Ye M. Li B.. (2018). Root exudate metabolites drive plant-soil feedbacks on growth and defense by shaping the rhizosphere microbiota. Nat. Commun. 9, 2738. doi: 10.1038/s41467-018-05122-7 30013066 PMC6048113

[B44] Huang W. He X. Y. Liu C. B. Li D. W. (2010). Effects of elevated carbon dioxide and ozone on foliar flavonoids of Ginkgo biloba. Adv. Mater Res. 113, 165–169. doi: 10.4028/www.scientific.net/AMR.113-116

[B45] Hussein R. A. El-Anssary A. A. (2019). Plants secondary metabolites: the key drivers of the pharmacological actions of medicinal plants. In Hebal Medicine. IntechOpen 13–30. doi: 10.5772/intechopen.76139

[B46] Hussin S. Geissler N. El-Far M. M. Koyro H. W. (2017). Effects of salinity and short-term elevated atmospheric CO2 on the chemical equilibrium between CO2 fixation and photosynthetic electron transport of Stevia rebaudiana Bertoni. Plant Physiol. Biochem. 118, 178–186. doi: 10.1016/j.plaphy.2017.06.017 28645057

[B47] Ibrahim M. H. Jaafar H. Z. (2012). Impact of elevated carbon dioxide on primary, secondary metabolites and antioxidant responses of Eleais guineensis Jacq.(Oil Palm) seedlings. Molecules 17, 5195–5211. doi: 10.3390/molecules17055195 22628041 PMC6268660

[B48] Idso S. B. Kimball B. A. Pettit G. R. III Garner L. C. Pettit G. R. Backhaus R. A. (2000). Effects of atmospheric CO2 enrichment on the growth and development of Hymenocallis littoralis (Amaryllidaceae) and the concentrations of several antineoplastic and antiviral constituents of its bulbs. Am. J. Bot. 87, 769–773. doi: 10.2307/2656884 10860907

[B49] Jaafar H. Z. Ibrahim M. H. Fakri N. F. M. (2012). Impact of soil field water capacity on secondary metabolites, phenylalanine ammonia-lyase (PAL), Maliondialdehyde (MDA) and photosynthetic responses of Malaysian Kacip Fatimah (Labisia pumila Benth). Molecules 17, 7305–7322. doi: 10.3390/molecules17067305 22695235 PMC6268701

[B50] Jamloki A. Bhattacharyya M. Nautiyal M. C. Patni B. (2021). Elucidating the relevance of high temperature and elevated CO2 in plant secondary metabolites (PSMs) production. Heliyon 7 (8), e07709. doi: 10.1016/j.heliyon.2021.e07709 34430728 PMC8371220

[B51] Jensen C. R. Mogensen V. O. Mortensen G. Fieldsend J. K. Milford G. F. J. Andersen M. N.. (1996). Seed glucosinolate, oil and protein contents of field-grown rape (Brassica napus L.) affected by soil drying and evaporative demand. Field Crops Res. 47, 93–105. doi: 10.1016/0378-4290(96)00026-3

[B52] Jochum G. M. Mudge K. W. Thomas R. B. (2007). Elevated temperatures increase leaf senescence and root secondary metabolite concentrations in the understory herb *Panax quinquefolius* (Araliaceae). Am. J. Bot. 94, 819–826. doi: 10.3732/ajb.94.5.819 21636451

[B53] Jordan D. N. Green T. H. Chappelka A. H. Lockaby B. G. Meldahl R. S. Gjerstad D. H. (1991). Response of total tannins and phenolics in loblolly pine foliage exposed to ozone and acid rain. J. Chem. Ecol. 17, 505–513. doi: 10.1007/BF00982121 24258801

[B54] Kaundal M. Bhatt V. Kumar R. (2018). Elevated CO2 and temperature effect on essential oil content and composition of *Valeriana jatamansi* Jones. with organic manure application in a Western Himalayan region. J. Essential Oil-Bearing Plants 21, 1041–1050. doi: 10.1080/0972060X.2018.1497547

[B55] Kaur T. Kumar A. Koul S. Bhat R. Bindu K. Bhat H. A.. (2015). Physiochemical and antioxidant profiling of *Salvia sclarea* L. at different climates in northwestern Himalayas. Acta Physiol. Plant 37, 132. doi: 10.1007/s11738-015-1879-7

[B56] Khalid K. A. (2006). Influence of water stress on growth, essential oil, and chemical composition of herbs (Ocimum sp). Int. Agrophys. 20 (40), 289–296.

[B57] Khare S. Singh N. B. Singh A. Hussain I. Niharika K. M. Yadav V.. (2020). Plant secondary metabolites synthesis and their regulations under biotic and abiotic constraints. J. Plant Biol. 63, 203–216. doi: 10.1007/s12374-020-09245-7

[B58] Kleinwächter M. Selmar D. (2015). New insights explain that drought stress enhances the quality of spice and medicinal plants: potential applications. Agron. Sustain. Dev. 35, 121–131. doi: 10.1007/s13593-014-0260-3

[B59] Kováčik J. Klejdus B. Hedbavny J. Bačkor M. (2009). Salicylic acid alleviates NaCl-induced changes in the metabolism of Matricaria chamomilla plants. Ecotoxicology 18, 544–554. doi: 10.1007/s10646-009-0312-7 19381803

[B60] Król A. Amarowicz R. Weidner S. (2015). The effects of cold stress on the phenolic compounds and antioxidant capacity of grapevine (Vitis vinifera L.) leaves. J. Plant Physiol. 189, 97–104. doi: 10.1016/j.jplph.2015.10.002 26555272

[B61] Ksouri R. Megdiche W. Debez A. Falleh H. Grignon C. Abdelly C. (2007). Salinity effects on polyphenol content and antioxidant activities in leaves of the halophyte Cakile maritima. Plant Physiol. Biochem. 45, 244–249. doi: 10.1016/j.plaphy.2007.02.001 17408958

[B62] Kumar R. Das S. Mishra M. Choudhury D. R. Sharma K. Kumari A.. (2021). Emerging roles of NAC transcription factor in medicinal plants: progress and prospects. 3 Biotech. 11, 1–14. doi: 10.1007/s13205-021-02970-x PMC8418584 34567930

[B63] Kumar D. Jha S. S. Kumar A. Singh S. K. (2021). “Omics and approaches in plant stress management,” In Kumar A. Droby S. (Eds.), Microbial Management of Plant Stresses (Cambridge, United Kingdom: Woodhead Publishing), pp. 107–117. doi: 10.1016/B978-0-323-85193-0.00003-6

[B64] Lavola A. Julkunen-Tiitto R. Pääkkönen E. (1994). Does ozone stress change the primary or secondary metabolites of birch (Betula pendula Roth.)? New Phytol. 126, 637–642. doi: 10.1111/j.1469-8137.1994.tb02959.x

[B65] Li Y. Kong D. Fu Y. Sussman M. R. Wu H. (2020). The effect of developmental and environmental factors on secondary metabolites in medicinal plants. Plant Physiol. Biochem. 148, 80–89. doi: 10.1016/j.plaphy.2020.01.006 31951944

[B66] Li Q. Lei S. Du K. Li L. Pang X. Wang Z.. (2016). RNA-seq based transcriptomic analysis uncovers α-linolenic acid and jasmonic acid biosynthesis pathways respond to cold acclimation in Camellia japonica. Sci. Rep. 6, 36463. doi: 10.1038/srep36463 27819341 PMC5098223

[B67] Lianopoulou V. Bosabalidis A. M. (2014). Traits of seasonal dimorphism associated with adaptation to cold stress in *Origanum dictamnus*L. *(*Lamiaceae). J. Biol. Res. Thessaloniki 21, 1–9. doi: 10.1186/2241-5793-21-17

[B68] Lianopoulou V. Bosabalidis A. M. Patakas A. Lazari D. Panteris E. (2014). Effects of chilling stress on leaf morphology, anatomy, ultrastructure, gas exchange, and essential oils in the seasonally dimorphic plant *Teucrium polium*(Lamiaceae). Acta Physiol. Plant. 36, 2271–2281. doi: 10.1007/s11738-014-1605-x

[B69] Lisar S. Y. S. Motafakkerazad R. Hossain M. M. Rahman I. M. M. (2012). “Water stress in plants: Causes, effects and responses”, In Rahman I. M. M. Hasegawa H. (Eds.), Water Stress pp. 1–14. *In Tech*. doi: 10.5772/39363

[B70] Liu H. Wang X. Wang D. Zou Z. Liang Z. (2011). Effect of drought stress on growth and accumulation of active constituents in *Salvia miltiorrhiza* Bunge. Ind. Crops Prod. 33, 84–88. doi: 10.1016/j.indcrop.2010.09.006

[B71] Lindberg J. Milliot J. Peethambaran B. Smith R. Nabbie F. Tettamanzi M. C. (2014). 14-3-3λ Affects Production of a Sinapoyl Derivative in Lignin Biosynthesis during Drought Stress in *Arabidopsis Thaliana* . Univ. J. Plant Sci. 2(4), 77–85. doi: 10.13189/ujps.2014.020401

[B72] Loreto F. Barta C. Brilli F. Nogues I. (2006). On the induction of volatile organic compound emissions by plants as consequence of wounding or fluctuations of light and temperature. Plant Cell Environ. 29, 1820–1828. doi: 10.1111/j.1365-3040.2006.01561.x 16913871

[B73] Ma C. H. Chu J. Z. Shi X. F. Liu C. Q. Yao X. Q. (2016). Effects of enhanced UV-B radiation on the nutritional and active ingredient contents during the floral development of medicinal chrysanthemum. J. Photochem. Photobiol. B: Biol. 158, 228–234. doi: 10.1016/j.jphotobiol.2016.02.019 26985737

[B74] Mabou F. D. Belinda I. Yossa N. (2021). TERPENES: Structural classification and biological activities. IOSR J. Pharm. Biol. Sci. 16 (3), 25–40. doi: 10.9790/3008-1603012540

[B75] Mahajan M. Kuiry R. Pal P. K. (2020). Understanding the consequence of environmental stress for accumulation of secondary metabolites in medicinal and aromatic plants. J. Appl. Res. Medicinal Aromatic Plants 18, 100255. doi: 10.1016/j.jarmap.2020.100255

[B76] Mahajan S. Tuteja N. (2005). Cold, salinity and drought stresses: an overview. Arch. Biochem. Biophys. 444, 139–158. doi: 10.1016/j.abb.2005.10.018 16309626

[B77] Mir B. A. Mir S. A. Khazir J. Tonfack L. B. Cowan D. A. Vyas D.. (2015). Cold stress affects antioxidative response and accumulation of medicinally important withanolides in *Withaniasomnifera* (L.) Dunal. Ind. Crops Prod. 74, 1008–1016. doi: 10.1016/j.indcrop.2015.06.012

[B78] Mirza H. Hossain M. A. Fujita M. (2010). Physiological and biochemical mechanisms of nitric oxide induced abiotic stress tolerance in plants. Am. J. Plant Physiol. 5, 295–324. doi: 10.3923/ajpp.2010.295.324

[B79] Mishra M. K. Chaturvedi P. Singh R. Singh G. Sharma L. K. Pandey V.. (2013). Overexpression of WsSGTL1 gene of *Withaniasomnifera*enhances salt tolerance, heat tolerance and cold acclimation ability in transgenic Arabidopsis plants. PloS One 8, e63064. doi: 10.1371/journal.pone.0063064 23646175 PMC3639950

[B80] Mishra T. (2016). Climate change and production of secondary metabolites in medicinal plants: a review. Int. J. Herb. Med. 4 (4), 27–30.

[B81] Mohammadi H. Ghorbanpour M. Brestic M. (2018). Exogenous putrescine changes redox regulations and essential oil constituents in field-grown Thymus vulgaris L. under well-watered and drought stress conditions. Ind. Crops Prod. 122, 119–132. doi: 10.1016/j.indcrop.2018.05.064

[B82] Naghiloo S. Movafeghi A. Delazar A. Nazemiyeh H. Asnaashari S. Dadpour M. R. (2012). Ontogenetic variation of total phenolics and antioxidant activity in roots, leaves and flowers of Astragalus compactus Lam. (Fabaceae). BioImpacts: BI 2 (2), 105–109. doi: 10.5681/bi.2012.015 23678448 PMC3648924

[B83] Naikoo M. I. Dar M. I. Raghib F. Jaleel H. Ahmad B. Raina A.. (2019). Role and regulation of plants phenolics in abiotic stress tolerance: An overview. Plant Signaling Mol. 5, 157–168. doi: 10.1016/B978-0-12-816451-8.00009-5

[B84] Najafi F. Khavari-Nejad R. A. (2010). The effects of salt stress on certain physiological parameters in summer savory (Satureja hortensis L.) plants. J. Stress Physiol. Biochem. 6, 13–21.

[B85] Neffati M. Marzouk B. (2008). Changes in essential oil and fatty acid composition in coriander (Coriandrum sativum L.) leaves under saline conditions. Ind. Crops Prod. 28, 137–142. doi: 10.1016/j.indcrop.2008.02.005

[B86] Ninou E. Paschalidis K. Mylonas I. (2017). Essential oil responses to water stress in greek oregano populations. J. Essential Oil Bear. Plants 20, 12–23. doi: 10.1080/0972060X.2016.1264278

[B87] Nowak M. Kleinwächter M. Manderscheid R. Weigel H.-J. Selmar D. (2010). Drought stress increases the accumulation of monoterpenes in sage (*Salvia officinalis*), an effect that is compensated by elevated carbon dioxide concentration. J. Appl. Bot. Food Qual. 83, 133–136.

[B88] Osman M. E. Elfeky S. S. El-Soud K. A. Hasan A. M. (2007). Response of Catharanthus roseus shoots to salinity and drought in relation to vincristine alkaloid content. Asian J. Plant Sci. 6 (8), 1223–1228. doi: 10.3923/ajps.2007.1223.1228

[B89] Oueslati S. Karray-Bouraoui N. Attia H. Rabhi M. Ksouri R. Lachaal M. (2010). Physiological and antioxidant responses of Mentha pulegium (Pennyroyal) to salt stress. Acta Physiol. Plant. 32, 289–296. doi: 10.1007/s11738-009-0406-0

[B90] Pant P. Pandey S. Dall’Acqua S. (2021). The influence of environmental conditions on secondary metabolites in medicinal plants: A literature review. Chem. Biodivers. 18, e2100345. doi: 10.1002/cbdv.202100345 34533273

[B91] Parida A. K. Das A. B. (2005). Salt tolerance and salinity effects on plants: a review. Ecotoxicol. Environ. Saf. 60, 324–349. doi: 10.1016/j.ecoenv.2004.06.010 15590011

[B92] Park J. S. Choung M. G. Kim J. B. Hahn B. S. Kim J. B. Bae S. C.. (2007). Genes up-regulated during red coloration in UV-B irradiated lettuce leaves. Plant Cell Rep. 26, 507–516. doi: 10.1007/s00299-006-0255-x 17086420

[B93] Park Y. J. Kwon D. Y. Koo S. Y. Truong T. Q. Hong S. C. Choi J.. (2023). Identification of drought-responsive phenolic compounds and their biosynthetic regulation under drought stress in Ligularia fischeri. Front. Plant Sci. 14, 1140509. doi: 10.3389/fpls.2023.1140509 36860897 PMC9968736

[B94] Paudel J. R. Amirizian A. Krosse S. Giddings J. Ismail S. A. A. Xia J.. (2016). Effect of atmospheric carbon dioxide levels and nitrate fertilization on glucosinolate biosynthesis in mechanically damaged Arabidopsis plants. BMC Plant Biol. 16, 1–12. doi: 10.1186/s12870-016-0752-1 27001610 PMC4802917

[B95] Paulsen J. Selmar D. (2016). Case study: the difficulty of correct reference values when evaluating the effects of drought stress: a case study with Thymus vulgaris. J. Appl. Bot. Food Qual. 89, 191–196. doi: 10.5073/JABFQ.2016.089.037.

[B96] Pellegrini E. Campanella A. Cotrozzi L. Tonelli M. Nali C. Lorenzini G. (2018). Ozone primes changes in phytochemical parameters in the medicinal herb Hypericum perforatum (St. John’s wort). Ind. Crops Prod. 126, 119–128. doi: 10.1016/j.indcrop.2018.10.002

[B97] Pellegrini E. Francini A. Lorenzini G. Nali C. (2015). Ecophysiological and antioxidant traits of Salvia officinalis under ozone stress. Environ. Sci. pollut. Res. 22, 13083–13093. doi: 10.1007/s11356-015-4569-5 25925147

[B98] Perrone A. Martinelli F. (2020). Plant stress biology in epigenomic era. Plant Sci. 294, 110376. doi: 10.1016/j.plantsci.2019.110376 32234231

[B99] Petropoulos S. A. Daferera D. Polissiou M. G. Passam H. C. (2008). The effect of water deficit stress on the growth, yield and composition of essential oils of parsley. Scientia Hortic. 115, 393–397. doi: 10.1016/j.scienta.2007.10.008

[B100] Punetha A. Kumar D. Suryavanshi P. Padalia R. C. Venkatesha K. T. (2022). Environmental abiotic stress and secondary metabolites production in medicinal plants: a review. J. Agric. Sci. 28, 351–362. doi: 10.15832/ankutbd.999117

[B101] Qian H. Xu Z. Cong K. Zhu X. Zhang L. Wang J.. (2021). Transcriptomic responses to drought stress in Polygonatumkingianum tuber. BMC Plant Biol. 21, 1–20. doi: 10.1186/s12870-021-03297-8 34781887 PMC8591914

[B102] Qiang Q. Gao Y. Yu B. Wang M. Ni W. Li S.. (2020). Elevated CO2 enhances growth and differentially affects saponin content in Paris polyphylla var. yunnanensis. Ind. Crops Prod. 147, 112124. doi: 10.1016/j.indcrop.2020.112124

[B103] Radušienė J. Karpavičienė B. Stanius Ž. (2012). Effect of external and internal factors on secondary metabolites accumulation in St. John’s worth. Botanica Lithuanica 18, 101–108. doi: 10.2478/v10279-012-0012-8

[B104] Rahimi S. Hasanloo T. (2016). The effect of temperature and pH on biomass and bioactive compound production in Silybum marianum hairy root cultures. Res. J. Pharmacogn. 3, 53–59.

[B105] Rahman A. (2013). Auxin: a regulator of cold stress response. Physiol. Plant. 147, 28–35. doi: 10.1111/j.1399-3054.2012.01617.x 22435366

[B106] Rastogi S. Shah S. Kumar R. Vashisth D. Akhtar M. Q. Kumar A.. (2019). Ocimum metabolomics in response to abiotic stresses: Cold, flood, drought and salinity. PloS One 14, e0210903. doi: 10.1371/journal.pone.0210903 30726239 PMC6364901

[B107] Razmjoo K. H. O. R. S. H. I. D. Heydarizadeh P. A. R. I. S. A. Sabzalian M. R. (2008). Effect of salinity and drought stresses on growth parameters and essential oil content of Matricaria chamomile. Int. J. Agric. Biol. 10, 451–454.

[B108] Razzaq M. K. Aleem M. Mansoor S. Khan M. A. Rauf S. Iqbal S.. (2021). Omics and CRISPR-Cas9 approaches for molecular insight, functional gene analysis, and stress tolerance development in crops. Int. J. Mol. Sci. 22, 1292. doi: 10.3390/ijms22031292 33525517 PMC7866018

[B109] Reifenrath K. Müller C. (2007). Species-specific and leaf-age dependent effects of ultraviolet radiation on two Brassicaceae. Phytochemistry 68, 875–885. doi: 10.1016/j.phytochem.2006.12.008 17257632

[B110] Ruelland E. Vaultier M. N. Zachowski A. Hurry V. (2009). Cold signalling and cold acclimation in plants. Adv. Bot. Res. 49, 35–150. doi: 10.1016/S0065-2296(08)00602-2

[B111] Said-Al Ahl H. A. H. Omer E. A. (2011). Medicinal and aromatic plants production under salt stress. A review. Herba olonica 57 (1), 72–87.

[B112] Sampaio B. L. Edrada-Ebel R. Da Costa F. B. (2016). Effect of the environment on the secondary metabolic profile of Tithonia diversifolia: a model for environmental metabolomics of plants. Sci. Rep. 6, 29265. doi: 10.1038/srep29265 27383265 PMC4935878

[B113] Santos A. P. Serra T. Figueiredo D. D. Barros P. Lourenço T. Chander S.. (2011). Transcription regulation of abiotic stress responses in rice: a combined action of transcription factors and epigenetic mechanisms. Omics: J. Integr. Biol. 15, 839–857. doi: 10.1089/omi.2011.0095 22136664

[B114] Sevillano L. Sanchez-Ballesta M. T. Romojaro F. Flores F. B. (2009). Physiological, hormonal and molecular mechanisms regulating chilling injury in horticultural species. Postharvest technologies applied to reduce its impact. J. Sci. Food Agric. 89, 555–573. doi: 10.1002/jsfa.v89:4

[B115] Shakeri A. Sahebkar A. Javadi B. (2016). Melissa officinalis L.–A review of its traditional uses, phytochemistry and pharmacology. J. Ethnopharmacol. 188, 204–228. doi: 10.1016/j.jep.2016.05.010 27167460

[B116] Shi L. Cui X. Shen Y. (2024). The roles of histone methylation in the regulation of abiotic stress responses in plants. Plant Stress 11, 100303. doi: 10.1016/j.stress.2023.100303

[B117] Shibata M. Amano M. Kawata J. Uda M. (1988). “Breeding process and characteristics of” summer queen”, a spray-type chrysanthemum cultivar for summer production,” in Bulletin of the National Research Institute of Vegetables, Ornamental Plants and Tea. Series A. 2 (A), 245–255.

[B118] Shil S. Dewanjee S. (2022). Impact of drought stress signals on growth and secondary metabolites (SMs) in medicinal plants. J. Phytopharmacol. 11 (5), 371–376. doi: 10.31254/phyto.2022.11511

[B119] Shohael A. M. Ali M. B. Yu K. W. Hahn E. J. Paek K. Y. (2006). Effect of temperature on secondary metabolites production and antioxidant enzyme activities in Eleutherococcussenticosus somatic embryos. Plant Cell Tissue Organ Cult. 85, 219–228. doi: 10.1007/s11240-005-9075-x

[B120] Snow M. D. Bard R. R. Olszyk D. M. Minster L. M. Hager A. N. Tingey D. T. (2003). Monoterpene levels in needles of Douglas fir exposed to elevated CO2 and temperature. Physiol. Plant. 117, 352–358. doi: 10.1034/j.1399-3054.2003.00035.x 12654035

[B121] Spitaler R. Schlorhaufer P. D. Ellmerer E. P. Merfort I. Bortenschlager S. Stuppner H. (2006). Altitudinal variation of secondary metabolite profiles in flowering heads of Arnica montana cv. ARBO Phytochem. 67, 409–417. doi: 10.1016/j.phytochem.2005.11.018 16405933

[B122] Stuhlfauth T. Klug K. Fock H. P. (1987). The production of secondary metabolites by Digitalis lanata during CO2 enrichment and water stress. Phytochemistry 26, 2735–2739. doi: 10.1016/S0031-9422(00)83581-5

[B123] Sun Y. Alseekh S. Fernie A. R. (2023). Plant secondary metabolic responses to global climate change: a meta-analysis in medicinal and aromatic plants. Global Change Biol. 29, 477–504. doi: 10.1111/gcb.16484 36271675

[B124] Sun L. Su H. Zhu Y. Xu M. (2012). Involvement of abscisic acid in ozone-induced puerarin production of Pueraria thomsnii Benth. suspension cell cultures. Plant Cell Rep. 31, 179–185. doi: 10.1007/s00299-011-1153-4 21947422

[B125] Szabó B. Tyihák E. Szabó G. Botz L. (2003). Mycotoxin and drought stress induced change of alkaloid content of Papaver somniferum plantlets. Acta Botanica Hungarica 45, 409–417. doi: 10.1556/ABot.45.2003.3-4.15

[B126] Tabatabaie S. J. Nazari J. (2007). Influence of nutrient concentrations and NaCl salinity on the growth, photosynthesis, and essential oil content of peppermint and lemon verbena. Turkish J. Agric. Forest. 31, 245–253.

[B127] Takshak S. Agrawal S. Á. (2014). Secondary metabolites and phenylpropanoid pathway enzymes as influenced under supplemental ultraviolet-B radiation in WithaniasomniferaDunal, an indigenous medicinal plant. J. Photochem. Photobiol. B: Biol. 140, 332–343. doi: 10.1016/j.jphotobiol.2014.08.011 25226342

[B128] Takshak S. Agrawal S. B. (2015). Defence strategies adopted by the medicinal plant Coleus forskohlii against supplemental ultraviolet-B radiation: Augmentation of secondary metabolites and antioxidants. Plant Physiol. Biochem. 97, 124–138. doi: 10.1016/j.plaphy.2015.09.018 26461242

[B129] Thirumurugan D. Cholarajan A. Raja S. S. Vijayakumar R. (2018). An introductory chapter: secondary metabolites. Secondary metabolites-sources Appl. 1, 1–13. doi: 10.5772/intechopen.79766

[B130] Tisserat B. R. E. N. T. Vaughn S. F. (2001). Essential oils enhanced by ultra-high carbon dioxide levels from Lamiaceae species grown *in vitro* and in *vivo* . Plant Cell Rep. 20, 361–368. doi: 10.1007/s002990100327

[B131] Ullrich S. F. Rothauer A. Hagels H. Kayser O. (2017). Influence of light, temperature, and macronutrients on growth and scopolamine biosynthesis in Duboisia species. Planta Med. 83, 937–945. doi: 10.1055/s-0043-106435 28371944

[B132] Upchurch R. G. (2008). Fatty acid unsaturation, mobilization, and regulation in the response of plants to stress. Biotechnol. Lett. 30, 967–977. doi: 10.1007/s10529-008-9639-z 18227974

[B133] Valentovic P. Luxova M. Kolarovic L. Gasparikova O. (2006). Effect of osmotic stresson compatible solutes content, membrane stability and water relations in twomaize cultivars. Plant Soil Environ. 52, 186–191. doi: 10.17221/3364-PSE

[B134] Varshney K. A. Gangwar L. P. Goel N. (1988). Choline and betaine accumulation in Trifolium alexandrinum L. during salt stress. Egypt J. Bot. 31, 81–86.

[B135] Verma N. Shukla S. (2015). Impact of various factors responsible for fluctuation in plant secondary metabolites. J. Appl. Res. Medicinal Aromatic Plants 2, 105–113. doi: 10.1016/j.jarmap.2015.09.002

[B136] Vuolo M. M. Lima V. S. Junior M. R. M. (2019). “Phenolic compounds: Structure, classification, and antioxidant power,” in Segura Campos M. R. (Ed.), Bioactive compounds (Cambridge, United Kingdom: Woodhead Publishing), pp. 33–50. doi: 10.1016/B978-0-12-814774-0.00002-5

[B137] Wang D. H. Du F. Liu H. Y. Liang Z. S. (2010). Drought stress increases iridoid glycosides biosynthesis in the roots of Scrophularianingpoensis seedlings. J. Med. Plants Res. 4, 2691–2699. doi: 10.5897/JMPR09.338

[B138] Wink M. (2010). “Introduction: Biochemistry, physiology and ecological functions of secondary metabolites,” In Wink M. (Ed.) Annual Plant Reviews, Volume 40: Biochemistry of plant secondary metabolism, pp. 1–19. doi: 10.1002/9781444320503.ch1

[B139] Wu W. Xu C. Liu X. (2018). “Climate change projections in the twenty-first century,” in Atlas of Environmental Risks Facing China Under Climate Change, pp. 15–30. IHDP/Future Earth–Integrated Risk Governance Project Series. Springer, Singapore. doi: 10.1007/978-981-10-4199-0_2

[B140] Xu Y. H. Liao Y. C. Zhang Z. Liu J. Sun P. W. Gao Z. H.. (2016). Jasmonic acid is a crucial signal transducer in heat shock induced sesquiterpene formation in Aquilaria sinensis. Sci. Rep. 6, 21843. doi: 10.1038/srep21843 26902148 PMC4763180

[B141] Yadav S. K. (2010). Cold stress tolerance mechanisms in plants. A review. Agron. Sustain. Dev. 30, 515–527. doi: 10.1051/agro/2009050

[B142] Yeshi K. Crayn D. Ritmejerytė E. Wangchuk P. (2022). Plant secondary metabolites produced in response to abiotic stresses has potential application in pharmaceutical product development. Molecules 27, 313. doi: 10.3390/molecules27010313 35011546 PMC8746929

[B143] Yuan Y. Tang X. Jia Z. Li C. Ma J. Zhang J. (2020). The effects of ecological factors on the main medicinal components of Dendrobium officinale under different cultivation modes. Forests 11, 94. doi: 10.3390/f11010094

[B144] Zeng Q. Feng L. Zhao C. Yang X. Yang R. Yin L.. (2008). Cloning of artemisinin biosynthetic cDNAs and novel ESTs and quantification of low temperature-induced gene overexpression. Sci. China C: Life Sci. 51 (3), 232–244. doi: 10.1007/s11427-008-0032-x 18246311

[B145] Zhang L. Wang Q. Guo Q. Chang Q. Zhu Z. Liu L.. (2012). Growth, physiological characteristics and total flavonoid content of Glechoma longituba in response to water stress. J. Medicinal Plants Res. 6, 1015–1024.

[B146] Zhang C. Yang D. Liang Z. Liu J. Yan K. Zhu Y.. (2019). Climatic factors control the geospatial distribution of active ingredients in Salvia miltiorrhiza Bunge in China. Sci. Rep. 9, 904. doi: 10.1038/s41598-018-36729-x 30696840 PMC6351527

[B147] Zhong J. J. Yoshida T. (1993). Effects of temperature on cell growth and anthocyanin production in suspension cultures of Perilla frutescens. J. Ferment. Bioeng. 76, 530–531. doi: 10.1016/0922-338X(93)90255-7

[B148] Zhou L. Li C. White J. F. Johnson R. D. (2021). Synergism between calcium nitrate applications and fungal endophytes to increase sugar concentration in *Festuca sinensis* under cold stress. PeerJ 9, e10568. doi: 10.7717/peerj.10568 35070512 PMC8759379

[B149] Zhu Y. Zhu G. Guo Q. Zhu Z. Wang C. Liu Z. (2013). A comparative proteomic analysis of Pinelliaternata leaves exposed to heat stress. Int. J. Mol. Sci. 14, 20614–20634. doi: 10.3390/ijms141020614 24132150 PMC3821634

[B150] Ziska L. H. Panicker S. Wojno H. L. (2008). Recent and projected increases in atmospheric carbon dioxide and the potential impacts on growth and alkaloid production in wild poppy (Papaver setigerum DC.). Clima. Change 91, 395–403. doi: 10.1007/s10584-008-9418-9

[B151] Zobayed S. M. A. Afreen F. Kozai T. (2007). Phytochemical and physiological changes in the leaves of St. John’s wort plants under a water stress condition. Environ. Exp. Bot. 59, 109–116. doi: 10.1016/j.envexpbot.2005.10.002

[B152] Zobayed S. S. P. K. Saxena P. K. (2004). Production of St. John’s wort plants under controlled environment for maximizing biomass and secondary metabolites. In Vitro Cell. Dev. Biol. Plant 40, 108–114. doi: 10.1079/IVP2003498

